# Effect of Mineral and Organic Fertilization on *desi* and *kabuli* Chickpea (*Cicer arietinum* L.): Plant Growth and Production, Hydration Properties, Bioactive Compounds, and Antioxidant Activity

**DOI:** 10.3390/plants10071441

**Published:** 2021-07-14

**Authors:** Antonella Pasqualone, Carmine Summo, Davide De Angelis, Giovanna Cucci, Davide Caranfa, Giovanni Lacolla

**Affiliations:** 1 Department of Soil, Plant, and Food Science (Di.S.S.P.A.), University of Bari ‘Aldo Moro’, Via Amendola, 165/A, I-70126 Bari, Italy; antonella.pasqualone@uniba.it (A.P.); carmine.summo@uniba.it (C.S.); davide.deangelis@uniba.it (D.D.A.); 2 Department of Agricultural and Environmental Science (Di.S.A.A.T.), University of Bari ‘Aldo Moro’, Via Amendola, 165/A, I-70126 Bari, Italy; davide.caranfa@gmail.com (D.C.); giovanni.lacolla@uniba.it (G.L.)

**Keywords:** pigmented chickpea, vegetal compost, 100 seed weight, hydration capacity, phenolic compounds, anthocyanins

## Abstract

Composting is a strategic technology to convert organic waste into environmentally friendly soil improvers, mitigating the pressure on landfills and contributing to sustainability. This research evaluates the effects of different doses of mineral/organic fertilizers on two chickpea types: *desi* and *kabuli*. A randomized block design with three replications and six conditions was adopted: non-fertilized control, two mineral fertilizations (M1, M2), and three organic fertilizations (B1, B2, B3). M1 and B1 provided for comparable NPK amounts. Fertilization and variety significantly influenced plant growth and production, and seed hydration. Fertilization had a lower influence on bioactive compounds. The highest seed yields were obtained with M2 (30–40–100 kg ha^−1^ of N, P_2_O_5_, and K_2_O, respectively. An addition of 40 kg ha^−1^ of P_2_O_5_ (M1) had no effect on seed yield. B1 (10 Mg ha^−1^ of Bio Vegetal) and M1 led to the same yield, which did not increase using higher doses of green compost. Mineral and organic fertilizations favored hydration and swelling of chickpeas. *Desi* chickpea showed a significantly higher seed yield but a lower seed weight than *kabuli*. Organic fertilization, combined with the recovery of peculiar chickpeas, which are more productive and richer in bioactive compounds, promotes a more sustainable food system.

## 1. Introduction

Chickpea (*Cicer arietinum* L.) is a widely cultivated legume, with a world production of around 14.3 million tons in 2019 [1]. According to their physic-chemical and genetic characteristics, chickpeas include two main categories: *kabuli*, a chickpea type having large beige seeds, and *desi*, with smaller and rough brown to black seeds [2]. Furthermore, in Apulia (Southern Italy), a subtype of black-colored chickpea with its own genetic features [3] is traditionally cultivated, but modern beige cultivars with a softer and easier to cook coat are progressively replacing it, causing a decrease in genetic diversity [4]. Recent studies, however, evidenced that the Apulian black subtype has an interesting potential for the development of food products, such as purée [5], or in mixture with wheat flour or semolina, various baked goods [6], and pasta [7]. Chickpeas are also the basis for the preparation of various traditional foods in the Mediterranean area and Middle East, such as *hummus*, as well as in India, where chickpea-based foods like *boondi*, *dhokla*, *pakora*, and *bhujia* are very popular [8], not to mention the numerous foods currently available on the market for vegetarian and vegan consumers.

Dietary habits are known to be closely linked to the onset of lifestyle-related pathologies. In this regard, the consumption of chickpeas leads to several health benefits, such as blood pressure regulation [9], decrease of postprandial glucose [10], and, in turn, prevention of diabetes mellitus and metabolic syndrome [11]. Chickpeas, indeed, have a good nutritional value, providing proteins, unsaturated fatty acids, dietary fiber, vitamins, and minerals [12,13]. Chickpeas also show significant antioxidant activity, related to the presence of bioactive compounds [13]. Interestingly, chickpea accessions with different contents of macronutrients and bioactive compounds showed a different ability to reduce the lipid over-accumulation in steatotic FaO hepatic cells and in mice liver [14]. In particular, black and brown *desi* type chickpeas showed a significantly higher content of anthocyanins and minerals (specifically Mn, Mg, and Ca), which were positively correlated with the antioxidant activity assessed with the DPPH assay [15].

Over than the genetic factors related to the chickpea variety, other factors could influence the composition of chickpeas, such as environmental and agronomic conditions. It is therefore important to also understand the effect of fertilization on the content of bioactive compounds and antioxidant activity of chickpea seeds. Several studies have been carried out to assess the influence of agronomic factors, mostly fertilization type and dose, on plant growth, physiological traits, and seed yield [16,17,18,19,20,21,22,23]. Other studies have considered the effect of mineral, organic, and biological fertilization on the chemical composition and physical characteristics of chickpea seeds [24,25]. However, no studies are available in the literature on the effect of fertilization on the content of bioactive compounds and the level of antioxidant activity of chickpeas.

To meet the needs of circular economy, composting is considered a strategic technology to convert organic waste into environmentally friendly soil improvers, mitigating the pressure on landfills and reducing the incineration of industrial and urban organic waste [26,27]. Composted organic amendments, obtained from a wide range of plant sources, are now considered effective means for increasing soil organic matter and restoring fertility [28]. Moreover, the demand for organic products is progressively increasing and the rules for organic productions impose the use of organic fertilizers with the exclusion of any synthetic product.

To contribute to a more sustainable agriculture and to the recycling of biomass of vegetable origin, the aim of this research was to evaluate the effects of different doses of mineral/organic fertilization on plant growth and production, hydration properties, bioactive compounds, and antioxidant activity of *desi* and *kabuli* chickpeas.

## 2. Results

Table 1 reports the effect of different doses of organic or mineral fertilization on plant height and yield parameters of chickpea. A significant effect of both fertilization treatment and chickpea variety on plant height, shoot dry biomass, seed yield (always *p* < 0.001), and 100 seed weight (*p* = 0.016) was observed. The effect of the treatment*variety interaction was not significant. Plant height of both types of chickpeas (*desi*, cv. Senise, and *kabuli*, cv. Sultano) increased significantly in the fertilized trials compared to the unfertilized control. The average increase accounted for 8% with mineral fertilization and 11% with organic fertilization (Bio Vegetal green compost). *Desi* chickpeas showed higher plant height than *kabuli*. However, no statistical difference was found after comparing different doses of organic fertilization among them. Additionally, with mineral fertilization, an increase in the dose of phosphorous did not result in a significant variation of plant height. The shoot dry biomass increased significantly in the fertilized trials compared to the unfertilized control. The variety of chickpea showed a significant effect, with the biomass of *desi* being higher than *kabuli.* The interaction between the treatment and the variety was not significant; therefore, the chickpea varieties under investigation responded in the same way to the fertilization. The mineral fertilization induced a significantly higher amount of shoot dry biomass than the organic one. No statistically significant differences between different doses of mineral or organic fertilization were observed for shoot dry biomass.

**Table 1:** Effect of different doses of mineral and organic fertilization on plant height and yield parameters of *desi* (cv. Senise) and *kabuli* (cv. Sultano) chickpeas (mean ± standard deviations).

Fertilization Type	Plant Height	Shoot Dry Biomass	Seed Yield	100 Seed Weight
	(cm)	(Mg ha^−1^)	(Mg ha^−1^)	g
***Desi* (cv. Senise)**				
Co	72.67 ± 2.23 ^cdef^	3.01 ± 0.16 ^e^	1.22 ± 0.15 ^bcde^	24.15 ± 0.52 ^c^
M1	79.34 ± 2.12 ^abcd^	3.75 ± 0.26 ^a^	1.38 ± 0.21 ^ab^	25.28 ± 0.23 ^c^
M2	77.00 ± 3.25 ^abcde^	3.68 ± 0.21 ^ab^	1.39 ± 0.19 ^a^	24.50 ± 0.31 ^c^
B1	83.00 ± 5.70 ^a^	3.16 ± 0.34 ^cde^	1.30 ± 0.22 ^abcd^	24.47 ± 0.22 ^c^
B2	82.00 ± 3.86 ^abc^	3.29 ± 0.28 ^cde^	1.32 ± 0.18 ^abc^	24.58 ± 0.21 ^c^
B3	82.33 ± 2.39 ^ab^	3.35 ± 0.49 ^bcde^	1.35 ± 0.34 ^abc^	24.85 ± 0.28 ^c^
***Kabuli* (cv. Sultano)**				
Co	64.34 ± 1.29 ^f^	2.64 ± 0.28^f^	0.97 ± 0.12 ^f^	28.41 ± 0.18 ^b^
M1	72.00 ± 3.21 ^def^	3.39 ± 0.21 ^abcd^	1.27 ± 0.22 ^abcde^	31.14 ± 0.57 ^ab^
M2	67.00 ± 1.45 ^f^	3.49 ± 0.34 ^abc^	1.25 ± 0.32 ^cde^	32.04 ± 0.62 ^a^
B1	68.00 ± 2.24 ^ef^	3.11 ± 0.29 ^de^	1.09 ± 0.11 ^ef^	32.51 ± 0.74 ^a^
B2	68.34 ± 2.39 ^ef^	3.20 ± 0.32 ^cde^	1.11 ± 0.14 ^de^	31.37 ± 0.42 ^ab^
B3	73.00 ± 2.40 ^bcdef^	3.01 ± 0.25 ^e^	1.12 ± 0.16 ^de^	31.19 ± 0.29 ^ab^
*p*-value T*V	0.285	0.115	0.721	0.066
*Desi*	79.39 ± 4.78 ^a^	3.37 ± 0.29 ^a^	1.33 ± 0.06 ^a^	24.65 ± 0.47 ^b^
*Kabuli*	68.83 ± 3.81 ^b^	3.14 ± 0.31 ^b^	1.13 ± 0.11 ^b^	31.11 ± 1.81 ^a^
*p*-value *V*	<0.001	<0.001	<0.001	<0.001
Co	68.50 ± 4.68 ^c^	2.82 ± 0.21 ^c^	1.09 ± 0.15 ^c^	26.28 ± 2.34 ^b^
M1	75.67 ± 4.76 ^ab^	3.57 ± 0.23 ^a^	1.31 ± 0.09 ^a^	28.21 ± 3.30 ^a^
M2	72.00 ± 5.76 ^bc^	3.59 ± 0.16 ^a^	1.29 ± 0.12 ^ab^	28.29 ± 4.22 ^a^
B1	75.5 ± 9.14 ^ab^	3.13 ± 0.05 ^b^	1.19 ± 0.12 ^b^	28.49 ± 4.46 ^a^
B2	75.33 ± 7.50 ^ab^	3.25 ± 0.17 ^b^	1.24 ± 0.11 ^ab^	27.97 ± 3.94 ^ab^
B3	77.67 ± 6.68 ^a^	3.18 ± 0.22 ^b^	1.24 ± 0.12 ^ab^	28.02 ± 3.69 ^ab^
*p*-value *T*	0.001	<0.001	<0.001	0.016

Co = unfertilized control, M = mineral fertilization, B = organic fertilization with Bio Vegetal green compost (Tersan Puglia, Modugno, Italy). Doses of fertilizers are reported in Table 2. Different letters in columns indicate significant differences according to the Tukey’s test at α = 0.05.

**Table 2:** Fertilization conditions applied to *desi* (cv Senise) and *kabuli* (cv Sultano) chickpeas.

Treatment	Biovegetal (Mg ha^−1^)	N (kg ha^−1^)	P_2_O_5_ (kg ha^−1^)	K_2_O (kg ha^−1^)
Co	0	0	0	0
M1	0	30 (urea)	80 (simple superphosphate)	100 (potassium sulfate)
M2	0	30 (urea)	40 (simple superphosphate)	100 (potassium sulfate)
B1	10	160.0	68.8	112.0
B2	15	240.0	109.0	168.0
B3	20	320.0	137.6	224.0

Co = unfertilized control; M = mineral fertilization; B = organic fertilization with Bio Vegetal green compost (Tersan Puglia, Modugno, Italy).

The seed yield increased significantly in the trials submitted to mineral and organic fertilization compared to the unfertilized control. The interaction between the two variables was not significant, suggesting that the fertilization affected the yield regardless of the variety of the chickpea. By comparing the organic fertilization at the lowest dose (B1, i.e., 10 Mg ha^−1^ of Bio Vegetal) with the mineral fertilization M1, which provided approximately the same NPK amount, a higher seed yield was observed in the latter. Such a difference with M1 was reduced by increasing the dose of Bio Vegetal compost. As already observed for plant height and shoot dry biomass, for seed yield, no statistically significant differences between different doses of mineral or organic fertilization were found.

Considering the “treatment” variable, significantly higher 100 seed weight was observed only for M1, M2, and B1 compared to the unfertilized control, whereas no significant differences were observed between different doses of mineral or organic fertilization.

Regarding the effect of chickpea variety on plant growth and yield parameters, *desi* chickpea showed a significantly greater plant height and shoot dry biomass than *kabuli*. Seed yield was also higher in *desi* than *kabuli* (1.33 Mg ha^−1^ and 1.13 Mg ha^−1^ on average for *desi* and *kabuli*, respectively), while the 100 seed weight was lower (24.65 g and 31.11 g on average for *desi* and *kabuli*, respectively).

The hydration and swelling properties of chickpea seeds (HC, HI, SC, and SI) were all significantly influenced (*p* < 0.001) by fertilization treatment and chickpea variety (Table 3). A significant effect of the treatment*variety interaction was observed only for SI (*p* = 0.012). HC, HI, SC, and SI increased significantly in the fertilized trials compared to the unfertilized control. No significant differences in HC and HI were observed between mineral and organic fertilization, at any of the doses considered, and both in *desi* and *kabuli* chickpeas. Regarding the effect of chickpea variety on the hydration properties, *desi* chickpeas showed a significantly lower ability to hydrate (0.28 vs. 0.34 g seed^−1^ for HC and 84.80 vs. 95.57 for HI in *desi* and *kabuli*, respectively) and swell (0.23 vs. 0.27 mL seed^−1^ for SC and 92.33 vs. 98.22 for SI in *desi* and *kabuli*, respectively) than *kabuli* ones. For SI, a significant interaction between the variables was observed. In particular, in *kabuli* chickpeas, a decrease of SI occurred when the concentration of the organic fertilization increased (B3), while no variation was observed in the *desi* variety.

Table 4 reports the effect of different doses of organic or mineral fertilization on the total phenolic compounds, total anthocyanin compounds, and antioxidant activity of chickpea seeds. A significant effect was exerted by the fertilization treatment on phenolic compounds (*p* < 0.001), but only in *kabuli* chickpeas. In detail, the phenolics of *kabuli* chickpeas tended to decrease with fertilization compared to the unfertilized control. No significant effect of fertilization was observed on the anthocyanin content (*p* = 0.064), with the only exception being the *desi* chickpeas submitted to M2 mineral fertilization, which contained significantly more anthocyanins than both the unfertilized chickpeas and those fertilized with Bio Vegetal at the B2 dose. No significant effect of the fertilization treatment was observed on the antioxidant activity of chickpeas (*p* = 0.063), irrespective of the variety.

Instead, both variety and treatment * variety interaction influenced the phenolics, anthocyanins, and antioxidant activity. A higher level of significance was observed for variety (*p* < 0.001) than for treatment * variety in the case of anthocyanins and antioxidant activity (*p* = 0.040 and *p* = 0.016, respectively). *Desi* chickpeas showed higher levels of phenolic compounds (1.29 mg ferulic acid/g d.m.) than *kabuli* chickpeas (1.24 mg ferulic acid/g d.m.). The strongest effect of variety was observed on total anthocyanins, which ranged from 12.11 to 13.28 mg cyanidin 3-*O*-glucoside /kg d.m. in *kabuli* and from 70.87 to 89.28 mg cyanidin 3-*O*-glucoside /kg d.m. in *desi* chickpeas. The antioxidant activity ranged from 1.08 to 1.18 μmol Trolox/g d.m. in *kabuli* and from 1.13 to 1.23 μmol Trolox/g d.m. in *desi* chickpeas.

## 3. Discussion

Mineral nutrition is one of the most important factors affecting plant growth and productivity, and N is the major nutrient required by crops. Both chickpea varieties were positively influenced by mineral fertilization (Table 1). An adequate supply of N is needed to achieve high yield potential in chickpea. Excessive doses of nitrogen can reduce production, while modest doses administered at sowing have a starter function [19,22,29]. Chickpea is usually managed with low fertilizer input. In agreement with our findings, Walley et al. [22] found that in dryland, mineral fertilization (30 or 45 kg N ha^−1^ and 40 kg P_2_O_5_ ha^−1^) enhanced the shoot dry biomass of both *desi* (cv. Myles) and *kabuli* (cv. Sanford) chickpeas. However, the same authors also observed that the seed yield significantly increased only in *desi*, while in our trial, only *kabuli* was positively influenced. Although high doses of phosphorous positively influence the production of chickpea [16], in our trials, an increase from 40 to 80 kg ha^−1^ of P_2_O_5_ did not lead to a significant increase in seed yield and 100 seed weight, in accordance with the findings of Bicer [30].

The organic fertilization induced an increase of the seed yield and 100 seed weight, but only in *kabuli*. Other authors reported positive effects on chickpea growth using different types of organic fertilizers (compost, vermicompost, farmyard manure), without specifying the chickpea type [16,31]. Compost can most likely improve plant growth through various mechanisms, including the reduction of nutritional constraints, improvement of soil water retention, and decreased incidence and impact of parasites [26].

Regarding the comparison between mineral and organic fertilization, we found no significant differences in plant height, seed yield, and 100 seed weight between M1 and B1 treatments, which provided approximately the same amount of nutrients. This absence of significant differences between the M1 mineral treatment and the B1 organic treatment is relevant to enhance the use of compost and to meet the needs of a circular economy. Our results can barely be compared with the existing literature due to differences in the specific doses or treatments. Seleiman and Abdelaal [25] compared mineral fertilization (35 kg N ha^−1^ as urea, 55 kg P_2_O_5_ ha^−1^, and 55 kg K_2_O ha^−1^) with an organic foliar treatment consisting of humic acid 2 kg ha^−1^ and found no significant differences in plant height, seed yield, and 100 seed weight. Chala and Obsa [16] observed that the combination of 1.75 Mg ha^−1^ of vermicompost with 50 kg ha^−1^ of phosphorus fertilizer led to a higher yield than with mineral fertilizer alone.

The positive influence of fertilization, compared to the non-fertilized control, on the hydration and swelling capacities of chickpeas (Table 3) agreed with Abdalla et al. [24], who found that mineral fertilization induced an increase in the hydration coefficient, which further increased in trials treated with biofertilizers (including *Rhizobium* and mycorrhizal inoculants). The HC values of *kabuli* chickpeas observed in our study agreed with those assessed by Patané et al. [32] in a set of 10 *kabuli* varieties. In detail, these authors found mean values of 0.36 g seed^−1^, very similar to our results (mean = 0.34 g seed^−1^).

The hydration properties of chickpea seeds were determined by soaking them in water for 7 h at room temperature. By soaking, chickpeas undergo an increase in weight, linked to the hydration capacity, and an increase in volume (swelling capacity). The soaking step is important both for domestic preparation and for the industrial production of canned chickpeas. Soaking reduces the cooking time necessary to obtain the desired softness, improves the sensory quality, and decreases the content of anti-nutritional factors, which can limit the biological value of chickpeas [33].

Khan et al. [34] and Olika et al. [35] found higher hydration and swelling capacity in *kabuli* than in *desi* chickpeas, and also pointed out that high values of these properties are related to shorter cooking times. The lower ability to hydrate and swell observed in *desi* compared to *kabuli* chickpeas was probably due to the harder seed coat of *desi* chickpeas, characterized by a rigid and extensively thickened palisade layer [2]. In addition, *desi* chickpeas are usually smaller and contain a lower amount of starch, sensible to swelling, than *kabuli* chickpeas [13]. Positive correlations were observed, in fact, between 100 seed weight and all hydration and swelling properties, at a higher level of significance for hydration than for swelling indices. In detail, 100 seed weight was correlated with HC (*r* = 0.879; *p* < 0.001), HI (*r* = 0.974; *p* < 0.001), SC (*r* = 0.742; *p* < 0.05), and SI (*r* = 0.766; *p* < 0.05).

Regarding the bioactive compounds (Table 4), the slight but significant effect of fertilization treatment on phenolics, observed only in *kabuli* chickpeas, induced a decrease of the content of these antioxidant molecules. The phenolic compounds are localized mostly in the pericarp of the seed, whose weight increase might have “diluted” their amount. A similar effect was already observed in faba beans [36]. A negative, but not significant, correlation between total phenolic compounds and 100 seed weight was observed.

Phenolic compounds are antioxidants that have been already reported in chickpeas by numerous authors [37,38,39]. The values of total phenolic compounds assessed in our trials roughly agreed with the 0.18–1.18 mg ferulic acid/g d.m. range observed in a collection of 57 accessions of *kabuli* and *desi* chickpeas [13]. Pigmented chickpea seeds, belonging to the *desi* type, are known to contain more total phenols than cream-colored and beige seeds of the *kabuli* type [40], in agreement with our results. The phenolic compounds identified in chickpeas have been reported to reduce chronic inflammatory responses [41,42].

Regarding anthocyanins, limited literature is available, but it is well established that the seeds of *desi* chickpea varieties contain more anthocyanins than *kabuli* [43]. The observed strong effect of chickpea variety on anthocyanins was therefore obvious, with anthocyanins being the red pigments responsible, at high doses, for the brown-to-black color of the seed coat typical of *desi* varieties [13]. The absence of a significant effect of fertilization on the anthocyanin content of *kabuli* chickpeas was also expected, due to a limited presence of these pigments in the *kabuli* type. A limited effect of fertilization was observed only in *desi* chickpea, where a more positive influence on anthocyanin content was induced by mineral than organic fertilization. No studies are reported in the literature on the effect of fertilization on the anthocyanin content of *desi* chickpeas, but a similar positive effect of fertilization has been reported in pigmented barley, where higher nitrogen levels induced an increase of anthocyanins in the culm and spike, and a decrease in the leaves [44]. The presence of anthocyanins in the leaves of *desi* chickpea, as well as in the basal part of the stem and in branches, has been reported as a consequence of stress induced by high temperatures (>35 °C) [45]. Being antioxidants, anthocyanins are part of the defense system of plants, and are known to mitigate abiotic and biotic stress [46]. Nutrient deficiencies, especially of phosphorus and nitrogen, have been reported to induce the accumulation of anthocyanins in the seedlings of many plant species, such as maize [47], cabbage, cauliflower, kohl rabi, radish, and canola [48].

The levels of anthocyanin compounds assessed in the trials were within the variation range (14.91–159.62 mg cyanidin 3-*O*-glucoside /kg d.m.) observed in a previous survey on a large collection of different chickpea genotypes [13]. Due to their antioxidant activity, anthocyanins have health-promoting properties with respect to cardiovascular disease, cancer, inflammation, and diabetes [49].

The observed values of antioxidant activity agreed with the ranges reported in the literature (1.16–3.39 μmol Trolox/g d.m.) [13]. The antioxidant activity mirrored the anthocyanin data. Therefore, pigmented chickpea seeds showed higher antioxidant activity, corroborating previous studies [40,50]. A positive and significant correlation (*r* = 0.769; *p* = 0.003) was found between the anthocyanins and antioxidant activity of chickpeas. Pigmented chickpeas could be a potentially functional food in consideration of their interesting antioxidant activity, able to prevent degenerative diseases associated with free radical damage.

## 4. Materials and Methods

### 4.1. Plant Cultivation and Experimental Trials

Chickpea (*Cicer arietinum* L.) cv. Sultano (*kabuli* type) and cv. Senise (*desi* type) were grown in 2019 at the experimental station of DISAAT Department of University of Bari ‘Aldo Moro’, sited in Bari, Italy, in a rotational crop system in alternation with durum wheat. The Senise cultivar belongs to the *desi* type and is characterized by a seed with a wrinkled surface that ends with a hook-shaped apex covered by a black integument, with creamy-colored cotyledons. This cultivar is usually cultivated in Puglia and Basilicata, Southern Italy. The Sultano cultivar belongs to the *kabuli* type, and is characterized by a larger smooth seed of regular shape with a light-colored integument.

The cultivation was carried out in cylindrical pots (0.72 m in diameter and 0.60 m high), filled with 293 kg of sandy-loam soil, whose main physic-chemical characteristics, determined according to Violante [51], are reported in Table 5.

A randomized block experimental design with three replications was adopted and six conditions were compared: non-fertilized control (Co), two mineral fertilizations, and three organic fertilizations, as reported in Table 2. The mineral fertilization consisted of nitrogen and potassium in the doses of 30 kg ha^−1^ of N, applied as urea, and 100 kg ha^−1^ of K_2_O, which are the quantities usually distributed to the chickpea crop in Southern Italy, while the doses of phosphorus, applied as simple superphosphate, accounted for 80 and 40 kg ha^−1^, indicated respectively with M1 and M2. Mineral fertilization was conducted at the time of sowing. The organic fertilization was based on the use of an organic fertilizer obtained from pruning residues of parks and gardens away from areas with high car traffic and wet organic municipal waste, all composted, namely the “Bio Vegetal” green compost (Tersan Puglia, Modugno, Italy).

The main physic-chemical characteristics of Bio Vegetal, determined according to the official methods of the current Italian legislation on organic fertilizers [52], are reported in Table 6. The green compost was used at the following doses: 10 (B1), 15 (B2), and 20 (B3) Mg ha^−1^. The dose of 10 Mg ha^−1^ of Bio Vegetal corresponded approximately to the same quantities of N, P_2_O_5_, and K_2_O provided by the highest dose of mineral fertilization (M1).

The compost was distributed in the 0–20 cm layer of soil, one month before sowing the chickpeas.

Sowing was carried out on 11 February 2019, at a density of 35 seeds m^−2^, which were distributed over 2 rows per pot. During the chickpea crop cycle, 410 mm of rain fell with a uniform distribution; therefore, no emergency irrigation interventions were necessary.

At the time of harvest, carried out when the grains were fully ripe on 21 July 2019, the following morpho-physiological parameters were measured: plant height and shoot dry biomass. Subsequently, on the dried chickpeas (12% humidity), the seed yield and 100 seed weight were determined.

### 4.2. Hydration Properties of Chickpea Seeds

The hydration properties of chickpea seeds were assessed according to the method described in Patané et al. [32], with a slight modification related to the soaking temperature, which was 20 °C instead of 30 °C. Water at room temperature was used to reproduce the usual domestic conditions adopted in the Mediterranean tradition for the preparation of legumes before cooking. First, the weight (Wi) of 50 chickpeas seeds was assessed. Then, the 50 chickpeas were transferred to a graduated cylinder and 100 mL of distilled water were added. Their initial volume (Vi) was determined, as (total volume −100 mL)/50 seeds. After 7 h, the water was removed, the seeds were dried with absorbent paper, and weighed again (Wf). Then, the final volume of the hydrated chickpeas (Vf) was determined by placing them again in a graduated cylinder and adding 100 mL of distilled water. A 7-h period of immersion in water was chosen as it was observed that maximum absorption occurs during the first 7 h of soaking [53]. The hydration capacity (HC) (g seed^−1^), representing the weight of the absorbed water per seed, was calculated as (Wf − Wi)/50, while the hydration index (HI) was calculated as (Wf − Wi)/Wi. The swelling capacity (SC) (mL seed^−1^), representing the increase in seed volume due to hydration, was calculated as (Vf − Vi)/50, while the swelling index (SI) was calculated as (Vf − Vi)/Vi. The analyses were carried out in triplicate.

### 4.3. Chickpea Milling

Dry chickpea seeds were milled to whole meal flour to determine the bioactive compounds and antioxidant activity. A laboratory mill equipped with a 1-mm sieve (Cyclotec Sample Mill, Tecator Foss, Hillerød, Denmark) was used.

### 4.4. Moisture Content Determination

The moisture content of whole meal flours was determined at 105 °C by means of an automatic moisture analyzer (Radwag Wagi Elektroniczne, Radom, Poland). The analysis was carried out in triplicate.

### 4.5. Determination of Total Phenolic Compounds

The total phenolic compounds (TPCs) were extracted from 1 g of whole meal flour of dry chickpea seeds with 10 mL of an aqueous-methanol solution (20:80 *v*/*v*). The suspension, put in centrifuge tubes, was stirred for 2 h in the dark, then centrifuged at 12,000× *g* for 3 min to recover the supernatant, which was subjected to Folin-Ciocalteu reaction in the conditions reported in Pasqualone et al. [54] and subsequent spectrophotometric measurement at 765 nm by a Cary 60 UV–Vis spectrophotometer (Agilent Technologies, Santa Clara, CA, USA). The content of TPC was expressed as mg g^−1^ of ferulic acid on dry matter (d.m.), considering a calibration curve previously prepared with ferulic acid (Merck KGaA, Darmstadt, Germany) at different concentrations. The analysis was carried out in triplicate.

### 4.6. Determination of Total Anthocyanin Compounds

The total anthocyanin compounds (TACs) were extracted from 1 g of whole meal flour of dry chickpea seeds with 10 mL of 85:15 (*v*/*v*) methanol/1 M HCl. The suspension was stirred for 30 min in the dark, then centrifuged at 12,000× *g* for 5 min to recover the supernatant. The pellet was re-extracted with 10 mL of the solvent in the same conditions and, after centrifugation at 12,000× *g* for 5 min, the two supernatants were mixed and their absorbance was determined at 535 nm by a Cary 60 UV–Vis spectrophotometer (Agilent Technologies, Santa Clara, CA, USA). Cyanidin 3-*O*-glucoside standard (Phytoplan, Heidelberg, Germany) was used to prepare the calibration curve in order to express total anthocyanins as mg kg^−1^ of cyanidin 3-*O*-glucoside on dry matter. The analysis was carried out in triplicate.

### 4.7. Determination of Antioxidant Activity

The same aqueous-methanol extract prepared for the extraction of TPC was used for determining the antioxidant activity (AA) by the 2,2-diphenyl-1-picrylhydrazyl (DPPH) radical scavenging capacity assay as described in Pasqualone et al. [55]. AA was then expressed as μmol (±)-6-Hydroxy-2,5,7,8-tetramethylchromane-2-carboxylic acid (Trolox; Merck KGaA, Darmstadt, Germany) equivalent g^−1^ on dry matter. The analysis was carried out in triplicate.

### 4.8. Statistical Analyses

All data were submitted to two-way ANOVA, at a significance level *α* = 0.05, followed by Tukey’s Honestly Significant Differences (HSD) test for post hoc multiple comparisons, in order to estimate the influence of each variable—fertilization treatment, chickpea variety—and of their first-order interaction, using Minitab 17 Statistical Software (Minitab, Inc., State College, PA, USA).

## 5. Conclusions

The obtained results highlight the significant effects of fertilization treatment and chickpea variety on plant growth and production, and the hydration properties of seeds. A lower influence of fertilization, instead, was observed on bioactive compounds, and no effect was assessed on the antioxidant activity.

The highest seed yields were obtained with mineral fertilization applied before sowing at the dose of 30–40–100 kg ha^−1^ of N, P_2_O_5_, and K_2_O, respectively (M2). A further contribution of 40 kg ha^−1^ of P_2_O_5_ (M1) did not result in an increase in seed yield. Good yields were also achieved with organic fertilization, at a dose of 10 Mg ha^−1^ (B1), of Bio Vegetal green compost distributed one month before sowing. This dose provided approximately the same NPK amount as the mineral fertilization M1. There was no increase in seed yield with the use of higher doses of green compost.

Both mineral and organic fertilization favored hydration and swelling in both types of chickpeas.

*Desi* chickpea, characterized by higher contents of bioactive compounds and antioxidant activity than *kabuli*, showed a significantly higher seed yield but lower 100 seed weight.

These results indicate that the organic fertilization with vegetal compost, combined with the recovery of pigmented varieties of chickpea, which are more productive and at the same time richer in bioactive compounds, are useful in creating more sustainable food systems suitable for modern dietary needs.

## Figures and Tables

**Table 3:** The effect of different doses of mineral and organic fertilization on the hydration and swelling properties of *desi* (cv. Senise) and *kabuli* (cv. Sultano) chickpeas (mean ± standard deviations).

Fertilization Type	Hydration Capacity	Hydration Index	Swelling Capacity	Swelling Index
	(g seed^−1^)		(mL seed^−1^)	
***Desi* (cv. Senise)**				
Co	0.24 ± 0.01 ^d^	79.01 ± 1.00 ^d^	0.18 ± 0.01 ^f^	84.19 ± 2.01 ^f^
M1	0.29 ± 0.02 ^c^	86.79 ± 1.01 ^bc^	0.24 ± 0.02 ^de^	95.21 ± 1.95 ^cde^
M2	0.27 ± 0.01 ^cd^	85.88 ± 1.32 ^bc^	0.25 ± 0.01 ^cde^	94.50 ± 2.00 ^cde^
B1	0.28 ± 0.01 ^cd^	86.61 ± 2.10 ^bc^	0.26 ± 0.02 ^bcd^	91.78 ± 2.29 ^e^
B2	0.29 ± 0.02 ^c^	85.93 ± 0.73 ^bc^	0.24 ± 0.01 ^de^	94.52 ± 0.20 ^cde^
B3	0.30 ± 0.01 ^bc^	84.58 ± 1.49 ^c^	0.23 ± 0.01 ^de^	93.81 ± 0.65 ^de^
***Kabuli* (cv. Sultano)**				
Co	0.27 ± 0.01 ^cd^	88.51 ± 1.36 ^b^	0.21 ± 0.02 ^ef^	91.93 ± 0.48 ^e^
M1	0.34 ± 0.02 ^ab^	96.92 ± 0.06 ^a^	0.27 ± 0.02 ^bcd^	98.12 ± 0.04 ^abc^
M2	0.36 ± 0.02 ^a^	97.83 ± 0.82 ^a^	0.29 ± 0.02 ^abc^	99.54 ± 0.17 ^ab^
B1	0.35 ± 0.01 ^a^	96.69 ± 0.54 ^a^	0.31 ± 0.01 ^a^	99.74 ± 0.11 ^ab^
B2	0.34 ± 0.02 ^ab^	95.92 ± 0.93 ^a^	0.30 ± 0.01 ^ab^	102.22 ± 2.35 ^a^
B3	0.36 ± 0.02 ^a^	97.55 ± 0.05 ^a^	0.27 ± 0.01 ^abcd^	97.80 ± 0.11 ^bcd^
*p*-value T*V	0.059	0.076	0.352	0.012
*Desi*	0.28 ± 0.02 ^b^	84.80 ± 2.99 ^b^	0.23 ± 0.03 ^b^	92.33 ± 4.16 ^b^
*Kabuli*	0.34 ± 0.03 ^a^	95.57 ± 3.38 ^a^	0.27 ± 0.04 ^a^	98.22 ± 3.35 ^a^
*p*-value *V*	<0.001	<0.001	<0.001	<0.001
Co	0.26 ± 0.02 ^b^	83.76 ± 5.31 ^b^	0.20 ± 0.02 ^c^	88.06 ± 4.44 ^c^
M1	0.32 ± 0.03 ^a^	91.85 ± 5.59 ^a^	0.25 ± 0.02 ^b^	96.66 ± 2.02 ^ab^
M2	0.32 ± 0.05 ^a^	91.85 ± 6.62 ^a^	0.27 ± 0.03 ^ab^	97.02 ± 3.04 ^ab^
B1	0.32 ± 0.04 ^a^	91.65 ± 5.69 ^a^	0.29 ± 0.03 ^a^	95.76 ± 4.59 ^b^
B2	0.32 ± 0.03 ^a^	90.92 ± 5.52 ^a^	0.27 ± 0.03 ^ab^	98.37 ± 4.48 ^a^
B3	0.33 ± 0.04 ^a^	91.07 ± 7.17 ^a^	0.25 ± 0.02 ^b^	95.80 ± 2.22 ^b^
*p*-value *T*	<0.001	<0.001	<0.001	<0.001

Co = unfertilized control, M = mineral fertilization, B = organic fertilization with Bio Vegetal green compost (Tersan Puglia, Modugno, Italy). Doses of fertilizers are reported in Table 2. Different letters in columns indicate significant differences according to the Tukey’s test at α = 0.05.

**Table 4:** The effect of different doses of mineral and organic fertilization on the total phenolic compounds, total anthocyanin compounds, and antioxidant activity of *desi* (cv. Senise) and *kabuli* (cv. Sultano) chickpeas (mean ± standard deviation).

Sample	Total Phenolic Compounds (mg ferulic acid/g d.m.)	Total Anthocyanins (mg cyanidin 3-*O*-glucoside/kg d.m.)	Antioxidant Activity (μmol Trolox/g d.m.)
***Desi* (cv. Senise)**			
Co	1.33 ± 0.05 ^ab^	71.43 ± 1.08 ^b^	1.23 ± 0.04 ^a^
M1	1.25 ± 0.03 ^bc^	76.02 ± 8.24 ^ab^	1.13 ± 0.01 ^ab^
M2	1.30 ± 0.09 ^abc^	89.28 ± 11.93 ^a^	1.22 ± 0.06 ^a^
B1	1.32 ± 0.03 ^abc^	74.73 ± 9.77 ^ab^	1.22 ± 0.08 ^a^
B2	1.29 ± 0.01 ^abc^	70.87 ± 0.42 ^b^	1.16 ± 0.01 ^ab^
B3	1.23 ± 0.01 ^bc^	78.79 ± 1.30 ^ab^	1.23 ± 0.04 ^a^
***Kabuli* (cv. Sultano)**			
Co	1.38 ± 0.01 ^a^	12.21 ± 1.41 ^c^	1.10 ± 0.00 ^b^
M1	1.12 ± 0.00 ^de^	12.74 ± 1.51 ^c^	1.08 ± 0.06 ^b^
M2	1.31 ± 0.02 ^abc^	12.11 ± 0.53 ^c^	1.13 ± 0.03 ^ab^
B1	1.22 ± 0.01 ^cd^	12.57 ± 0.10 ^c^	1.12 ± 0.01 ^ab^
B2	1.32 ± 0.04 ^abc^	13.28 ± 1.14 ^c^	1.18 ± 0.03 ^ab^
B3	1.07 ± 0.02 ^e^	12.75 ± 1.38 ^c^	1.08 ± 0.01 ^b^
*p*-value T*V	<0.001	0.040	0.016
*Desi*	1.29 ± 0.05 ^a^	76.85 ± 8.76 ^a^	1.20 ± 0.06 ^a^
*Kabuli*	1.24 ± 0.12 ^b^	12.61 ± 1.03 ^b^	1.11 ± 0.04 ^b^
*p*-value *V*	<0.001	<0.001	<0.001
Co	1.36 ± 0.04 ^a^	41.82 ± 32.46	1.16 ± 0.08
M1	1.19 ± 0.07 ^c^	44.38 ± 35.06	1.11 ± 0.05
M2	1.30 ± 0.06 ^ab^	50.70 ± 42.94	1.17 ± 0.07
B1	1.27 ± 0.06 ^b^	43.65 ± 34.60	1.17 ± 0.07
B2	1.31 ± 0.03 ^ab^	42.08 ± 31.56	1.17 ± 0.02
B3	1.15 ± 0.09 ^c^	45.77 ± 36.19	1.16 ± 0.08
*p*-value *T*	<0.001	0.064	0.063

Co = unfertilized control, M = mineral fertilization, B = organic fertilization with Bio Vegetal green compost (Tersan Puglia, Modugno, Italy). Doses of fertilizers are reported in Table 2. Trolox = (±)-6-Hydroxy-2,5,7,8-tetramethylchromane-2-carboxylic acid; T = Treatment; V = Variety. Different letters in column indicate significant differences according to the Tukey’s test at α = 0.05.

**Table 5:** Particle size distribution, chemical properties, and hydrologic properties of the soil used in the trials.

Parameter	Value
Particle size distribution:	
Total sand (2 > ∅ > 0.02 mm) (g kg^−1^)	506
Silt (%) (0.02 > ∅ > 0.002 mm) (g kg^−1^)	260
Clay (%) (∅ < 0.002 mm) (g kg^−1^)	234
Chemical properties:	
Total nitrogen (Kjeldahl method) (g kg^−1^)	1.2
Available phosphorus (Olsen method) (mg kg^−1^)	18.5
Exchangeable potassium (BaCl_2_ method) (mg kg^−1^)	231
Organic matter (Walkley Black method) (g 100 g^−1^)	1.7
Total limestone (g 100 g^−1^)	2.6
Active limestone (g 100 g^−1^)	4.6
pH	7.4
ECe (dS m^−1^)	0.5
ESP	0.7
CEC (BaCl_2_ method) (meq 100 g^−1^ of soil d.m.)	23.4
Hydrologic properties:	
Field capacity (g kg^−1^ of soil d.m.)	252
Wilting point (−1.5 MPa) (g kg^−1^ of soil d.m.)	145
Bulk density (t m^−3^)	13.6

ECe = saturation extract electrical conductivity; ESP = exchangeable sodium percentage; CEC = cation exchange capacity.

**Table 6:** Physic-chemical properties of Bio Vegetal green compost (Tersan Puglia, Modugno, Italy). All values are expressed as dry matter.

Parameter	Value
Moisture (g 100 g^−1^)	20
pH	7.5
ECe (dS m^−1^)	1.57
Total carbon (C) (g kg^−1^)	300
Total nitrogen (N) (g kg^−1^)	20
Organic nitrogen (g 100 g^−1^)	90
C/N	15
Total phosphorus (P) (g kg^−1^)	8.6
Total potassium (K) (g kg^−1^)	14
Calcium (Ca) (g kg^−1^)	3.5
Magnesium (Mg) (g kg^−1^)	1.2
Zinc (Zn) (mg kg^−1^)	164
Copper (Cu) (mg kg^−1^)	97

ECe = saturation extract electrical conductivity.

## Data Availability

The data that support the findings of this study are available from the corresponding author, upon reasonable request.
